# Characterization of Small Dosimeters Used for Measurement of Eye Lens Dose for Medical Staff during Fluoroscopic Examination

**DOI:** 10.3390/diagnostics11020150

**Published:** 2021-01-20

**Authors:** Kosuke Matsubara, Sayu Yoshida, Ayaka Hirosawa, Thunyarat Chusin, Yasushi Furukawa

**Affiliations:** 1 Department of Quantum Medical Technology, Faculty of Health Sciences, Institute of Medical, Pharmaceutical and Health Sciences, Kanazawa University, 5-11-80 Kodatsuno, Kanazawa, Ishikawa 920-0942, Japan; 2 Department of Radiology, Takaoka City Hospital, 4-1 Takaramachi, Takaoka, Toyama 933-8550, Japan; sayu0411snoopyy@gmail.com; 3 Department of Medical Technology, Toyama Prefectural Central Hospital, 2-2-78 Nishinagae, Toyama, Toyama 930-8550, Japan; ayakhiro@yahoo.co.jp; 4 Department of Quantum Medical Technology, Division of Health Sciences, Graduate School of Medical Sciences, Kanazawa University, 5-11-80 Kodatsuno, Kanazawa, Ishikawa 920-0942, Japan; y_furukawa1103@yahoo.co.jp; 5 Department of Radiological Technology, Faculty of Allied Health Sciences, Naresuan University, Muang, Phitsanulok 65000, Thailand; thunyaratc@nu.ac.th; 6 Department of Radiological Technology, Nagoya University Hospital, 65 Tsurumaicho, Showa, Nagoya 466-8560, Japan

**Keywords:** radiation protection, occupational exposure, eye lens, radiophotoluminescence glass dosimeter, optically stimulated luminescence dosimeter

## Abstract

This study aimed to evaluate the property of small dosimeters used for measuring eye lens doses for medical staff during fluoroscopic examination. Dose linearity, energy dependence, and directional dependence of scattered X-rays were evaluated for small radiophotoluminescence glass dosimeters (RPLDs), those with a tin filter (Sn-RPLDs), and small optically stimulated luminescence dosimeters (OSLDs). These dosimeters were pasted on radioprotective glasses, and accumulated air kerma was obtained after irradiating the X-rays to a patient phantom. Strong correlations existed between fluoroscopic time and accumulated air kerma in all types of dosimeters. The energy dependence of Sn-RPLD and OSLD was smaller than that of RPLD. The relative dose value of the OSLD gradually decreased as the angle of the OSLD against the scattered X-rays was larger or lower than the right angle in the horizontal direction. The ranges of relative dose values of RPLD and Sn-RPLD were larger than that of OSLD in the vertical direction. The OSLDs showed lower doses than the RPLDs and Sn-RPLDs, especially on the right side of the radioprotective glasses. These results showed that RPLDs, Sn-RPLDs, and OSLDs had different dosimeter properties, and influence measured eye lens doses for the physician, especially on the opposite side of the patient.

## 1. Introduction

Occupational dose limits are set to protect workers from the effects of ionizing radiation. The increased use of therapeutic and diagnostic radiology procedures results in larger doses for medical staff. Thus, individual monitoring of external radiation is essential in assessing occupational exposure. A new threshold dose of 0.5 Gy was suggested by the International Commission on Radiological Protection (ICRP) in 2011 for radiation effects on the eye lens, which is a highly radiosensitive tissue. Moreover, the ICRP recommended a new occupational equivalent dose limit for the eye lens of 20 mSv/year, averaged over defined periods of five years, with no single year exceeding 50 mSv [1]. Consequently, the International Atomic Energy Agency (IAEA) adopted the occupational dose limit for the eye lens in the IAEA Basic Safety Standards [2]. This new occupational dose limit for the eye lens is currently being introduced into the regulatory documents of several countries.

This new occupational dose limit may require regular monitoring of eye lens doses. Several studies have investigated the occupational exposure of the eye lens to radiation during procedures that employed fluoroscopy and showed that professionals who perform endoscopic retrograde cholangiopancreatography (ERCP) may exceed the revised eye lens dose limit when using an overcouch X-ray system [3,4]. Operators and other medical staff must stand close to the patient and face the risk of receiving high radiation doses to their eye lenses during ERCP [3,4].

The most accurate method for monitoring the equivalent dose to the lens of the eye is to measure the personal dose equivalent at 3 mm depth, Hp(3), with a dosimeter worn as close as possible to the eye and calibrated on a phantom representative of the head [5]. Dedicated eye lens dosimeters, such as Dosiris (IRSN, Fontenay-aux-Roses, France) [6,7,8,9], VISION (Landauer, Glenwood, IL, USA) [10], and EYE-D (RadCard, Krakow, Poland) [3,11], which are based on thermoluminescent materials, require service charges. Consequently, some previous studies showed the possibility of using small-type radiophotoluminescence glass dosimeters (RPLDs) or small-type optically stimulated luminescence dosimeters (OSLDs) for measuring occupational eye lens doses [12,13,14,15]. However, accurately responding to eye lens doses regardless of the type of dosimeters is crucial. Furthermore, Okazaki et al. [16] investigated the influence of directional and energy dependence of OSLDs on the measurement of the entrance surface dose of a patient during X-ray diagnosis and found that it was not significant. In these studies, X- or γ-ray beams were irradiated to the dosimeters that were set in air condition or placed on the surface of the cylindrical phantom. The response of these small dosimeters to scattered X-rays from patients needs to be investigated for a better understanding of the properties of RPLDs and OSLDs used for occupational eye lens dosimetry.

Based on these backgrounds, the present study aimed to evaluate the property of small dosimeters used for measuring eye lens doses for medical staff during fluoroscopic examination.

## 2. Materials and Methods

### 2.1. Radiographic and Fluoroscopic Equipment

An AXIOM Luminos dRF (Siemens Healthineers, Erlangen, Germany) was used as the radiographic and fluoroscopic equipment. It has an overcouch X-ray tube and additional copper filters with thicknesses of 0.1, 0.2, 0.3, and 0.4 mm. Users can choose one of the filters when taking radiography if filtration is needed. Moreover, a copper filter of 0.2 or 0.3 mm needs to be chosen when using fluoroscopy.

### 2.2. Dosimeters

RPLDs (GD-302M; AGC Techno Glass, Shizuoka, Japan), those with an energy compensation tin filter (Sn-RPLDs) (GD-352M; AGC Techno Glass), and OSLDs (nanoDot; Landauer) were used in this study.

The RPLD elements consisted of silver activated phosphate glass and had a length and diameter of 12 and 1.5 mm, respectively. Moreover, they were encapsulated in plastic holders, and only those of Sn-RPLDs had an energy compensation filter of 0.75-mm tin. The RPLD elements were annealed at 400 °C for 20 min before each exposure. After each exposure, they were further heated to 70 °C for 30 min and were read using an FGD-1000 reader (Chiyoda Technol, Tokyo, Japan) following the manufacturer’s recommended protocol. The reader was calibrated with a calibration RPLD exposed to a ^137^Cs γ-ray beam (662 keV). Furthermore, all RPLD elements were read five times, the read values were averaged before and after irradiation, and air kerma was obtained by subtracting the initial value from the final value.

The OSLDs consisted of a 0.3-mm-thick aluminum oxide-based (Al_2_O_3_: C) disk with a diameter of 5 mm (an active diameter of 4 mm), and were encapsulated in plastic cases. The OSLDs were initialized with fluorescent light before each exposure. Consequently, their dose values were read out using a microStar reader (Landauer) after each exposure. The reader was calibrated with a set of calibration OSLDs exposed to an 80-kV X-ray beam. All OSLDs were read five times, the read values were averaged before and after irradiation, and air kerma was obtained by subtracting the initial value from the final value.

A 180 cm^3^ general-purpose ionization chamber (10 × 6 − 180; Radcal, Monrovia, CA, USA) and a multifunction digitizer module (AGDM; Radcal) were used as a reference dosimeter to evaluate the energy dependence of RPLDs, Sn-RPLDs, and OSLDs to scattered X-rays (see Section 2.3.2).

### 2.3. Basic Characteristics of Small Dosimeters

**Figure 1:**
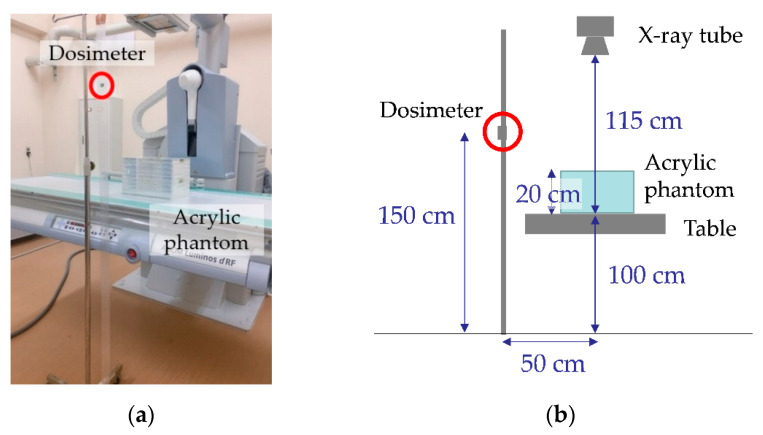
An experimental setup for evaluating the basic characteristics of small dosimeters. An acrylic block phantom with a thickness of 20 cm was placed at the center of the irradiation field. In addition, one radiophotoluminescence glass dosimeter (RPLD), that with an energy compensation tin filter (Sn-RPLD), or optically stimulated luminescence dosimeter (OSLD) was placed at a height of 150 cm from the floor and at a distance of 50 cm from the phantom: (**a**) photograph and (**b**) schematic of the experimental setup.

An acrylic block phantom with a thickness of 20 cm was placed at the center of the irradiation field, and one RPLD, Sn-RPLD, or OSLD was placed at a height of 150 cm from the floor and at a distance of 50 cm from the phantom to evaluate the basic characteristics of RPLDs, Sn-RPLDs, and OSLDs when measuring scattered X-rays (Figure 1).

#### 2.3.1. Dose Linearity

Primary X-rays were exposed to the phantom with a pulsed fluoroscopy mode for 5, 10, and 20 min with the following parameters: tube voltage (73 kV), tube current (40.3 mA), pulse width (7.2 ms), pulse rate (15 p/s), additional filter (0.2 mmCu), and irradiation field (20 cm × 20 cm) at the detector position. The same measurements were performed five times by replacing the exposed RPLD, Sn-RPLD, or OSLD with new ones after each exposure.

#### 2.3.2. Energy Dependence

The ionization chamber (reference dosimeter) was placed side by side with the RPLD, Sn-RPLD, or OSLD to evaluate the energy dependences when measuring scattered X-rays generated with different tube voltages and additional filters. For each combination of tube voltage and additional filter, primary X-rays were exposed to the phantom 10 times with a radiography mode with the following parameters: tube voltages (60, 70, 81, 90, 100, 109, and 121 kV; effective energies of the primary X-rays are shown in Table 1), a tube current–time product (10 mAs), additional filters (0.2 and 0.3 mmCu), and irradiation field (20 cm × 20 cm) at the image receptor position. The same measurements were performed five times by replacing the exposed RPLD, Sn-RPLD, or OSLD with new ones after 10 exposures. A sensitivity (S) analysis of the RPLD, Sn-RPLD, and OSLD relative to the reference dosimeter (S) was calculated using the following equation:

(1) $$S=\;\frac{M}{M_{\mathrm{ref}}\cdot k_{\mathrm{TP}}\cdot P_{\mathrm{ion}}}$$

where *M* represents measured air kerma obtained from RPLD, Sn-RPLD, or OSLD; *M*_ref_ represents that obtained from the reference dosimeter; *k*_TP_ represents temperature and pressure correction factor; and *P*_ion_ represents ion recombination correction factor (1.00).

#### 2.3.3. Directional Dependence

**Figure 2:**
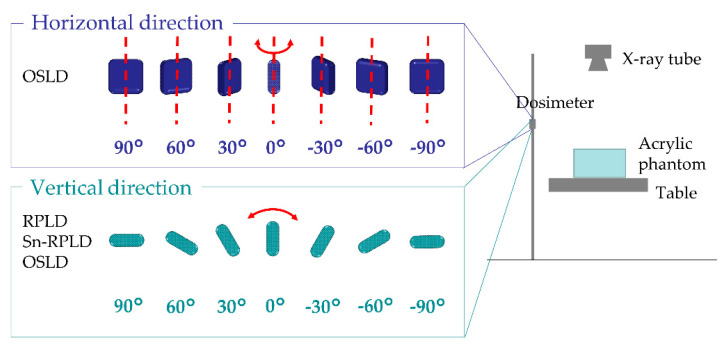
An experimental setup for evaluating the directional dependence of small dosimeters. The RPLD or Sn-RPLD was tilted vertically from −90° to 90° at intervals of 30° and the OSLD was tilted vertically from −90° to 90° at intervals of 30° and horizontally from −90° to 90° at intervals of 30°.

The RPLD or Sn-RPLD was tilted vertically from −90° to 90° at intervals of 30° and the OSLD was tilted vertically from −90° to 90° at intervals of 30° and horizontally from −90° to 90° at intervals of 30° (Figure 2). The RPLD and Sn-RPLD were not horizontally tilted because they were cylindrical. For each angle, primary X-rays were irradiated to the phantom with a pulsed fluoroscopy mode for 10 min with the following parameters: tube voltage (73 kV), tube current (40.3 mA), pulse width (7.2 ms), pulse rate (15 p/s), additional filter (0.2 mmCu), and irradiation field (20 cm × 20 cm) at the detector position. The same measurements were performed five times by replacing the exposed RPLD, Sn-RPLD, or OSLD with new ones after each exposure.

### 2.4. Eye Lens Dose Measurement That Simulated an ERCP Physician

**Figure 3:**
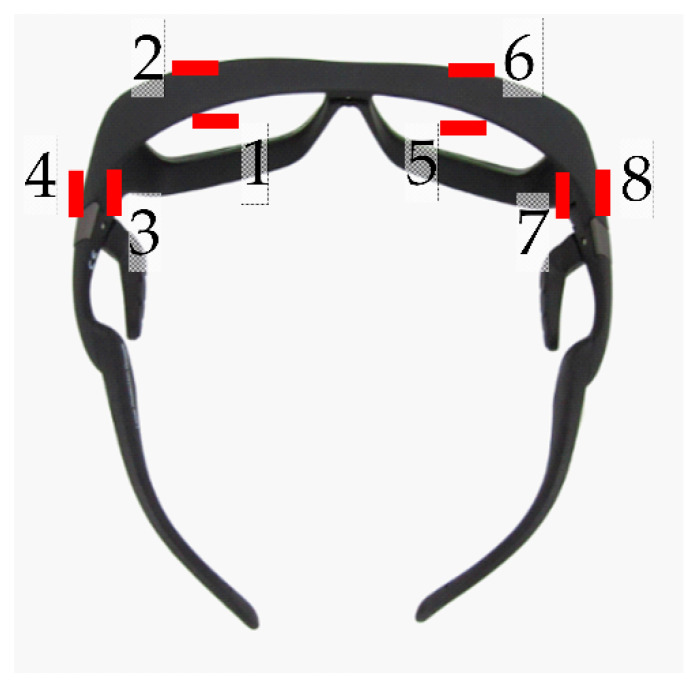
Locations of small dosimeters on radioprotective glasses: 1, left inside lens; 2, left outside lens; 3, left inside frame; 4, left outside frame; 5, right inside lens; 6, right outside lens; 7, right inside frame; and 8, right outside frame.

**Figure 4:**
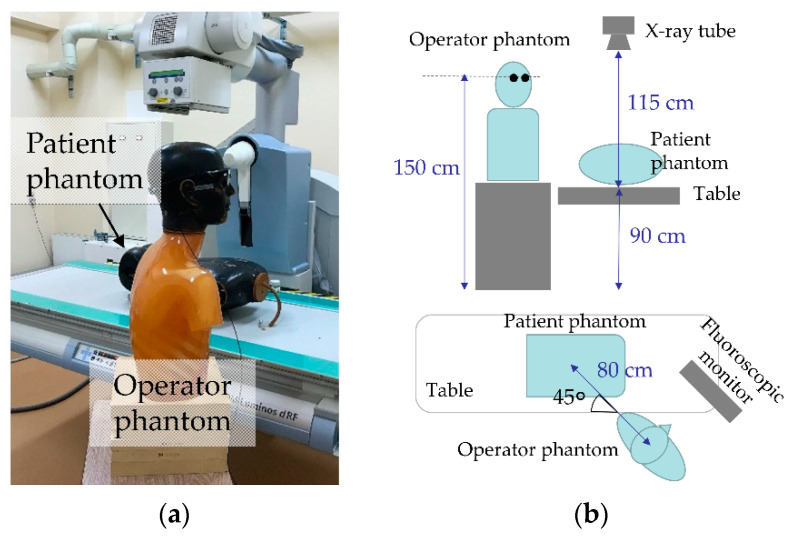
An experimental setup for eye lens dose measurement that simulated an ERCP physician. The operator phantom was turned toward a fluoroscopic monitor: (**a**) photograph and (**b**) schematic of the experimental setup.

RPLDs, Sn-RPLDs, or OSLDs were pasted at eight locations on radioprotective glasses (Dr. View X-RAY AF; KAZ, Fukui, Japan) with the protection of 0.85 mm lead equivalence measured at a 100 kV X-ray, including the inside lens, outside lens, inside frame, and outside frame of the left and right sides (Figure 3). Moreover, the glasses were placed on a phantom that simulated an ERCP physician as the main operator. The operator phantom was rotated 45° clockwise from 0°, where the position of 0° was defined as the physician facing the patient phantom, because operators generally turn toward a fluoroscopic monitor during fluoroscopy (Figure 4).

A body phantom that simulated a patient was placed at the center of the irradiation field, and primary X-rays were irradiated to the phantom with a pulsed fluoroscopy mode for 10 min with the following parameters that were designed for ERCP: tube voltage (73 kV), tube current (43.0 mA), pulse width (7.2 ms), pulse rate (15 p/s), additional filter (0.2 mmCu), and irradiation field (22.3 cm × 22.3 cm) at the detector position. No additional X-rays were exposed to the phantom with a radiography mode. The same measurements were performed five times by replacing the exposed RPLDs, Sn-RPLDs, or OSLDs with new ones after each exposure. Air kerma (*K*_a_) was corrected by the following equation:

(2) $$K_{a}=\;M\cdot \frac{1}{S}$$

where *M* represents the measured air kerma from RPLD, Sn-RPLD, or OSLD and *S* represents sensitivity at 73-kV X-rays with a 0.2 mmCu filter calculated by Equation (1). The sensitivity of each RPLD, Sn-RPLD, or OSLD was obtained beforehand by the same geometrical setup as described in Section 2.3.2.

### 2.5. Statistical Analysis

All statistical analyses were performed using commercially available software (SPSS Statistics 25; IBM, Armonk, NY, USA). A one-way analysis of variance test was used to compare the differences in air kerma among groups. Moreover, Tukey’s test was used for post hoc analysis. A *p* value of <0.05 was considered statistically significant.

## 3. Results

### 3.1. Dose Linearity

**Figure 5:**
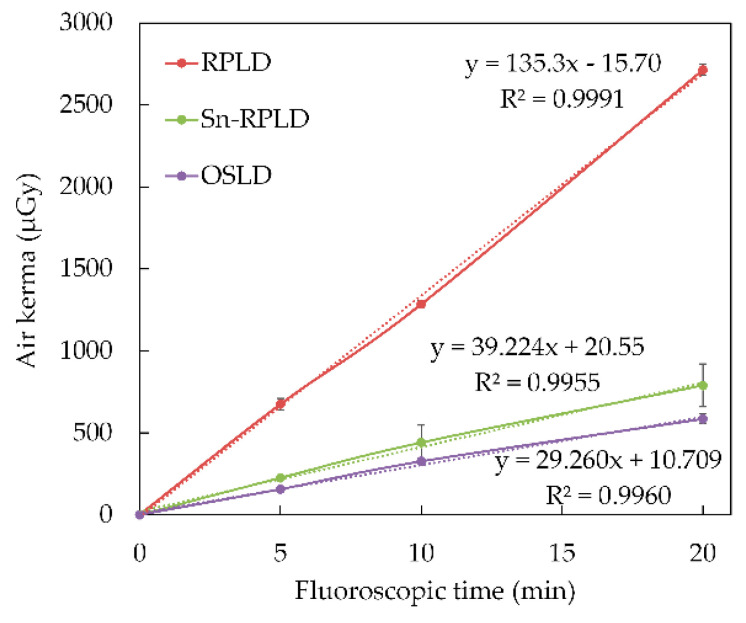
The accumulated air kerma of small dosimeters as a function of fluoroscopic time.

Although the accumulated air kerma measured by the RPLDs was approximately four times greater than that measured by the Sn-RPLDs and OSLDs, strong correlations were noted between fluoroscopic time and accumulated air kerma measured by RPLDs (R^2^ = 0.9991), fluoroscopic time and Sn-RPLDs (R^2^ = 0.9955), and fluoroscopic time and OSLDs (R^2^ = 0.9960), as shown in Figure 5. The coefficients of variation for the accumulated air kerma measured by RPLDs, Sn-RPLDs, and OSLDs were 0.012–0.053, 0.042–0.201, and 0.052–0.090, respectively.

### 3.2. Energy Dependence

**Figure 6:**
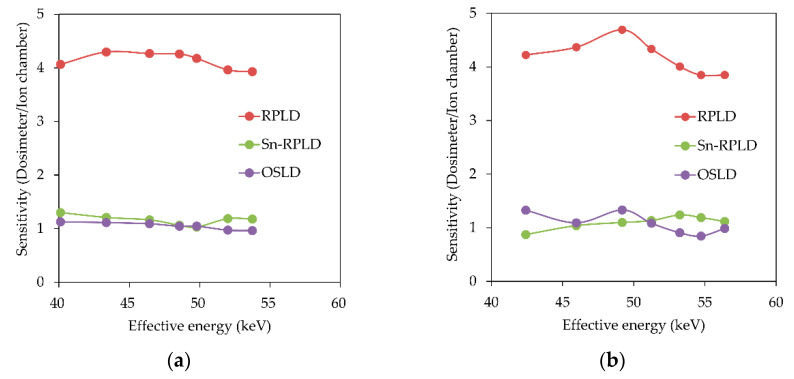
Sensitivity of small dosimeters as a function of effective energy: (**a**) additional filter of 0.2 mmCu and (**b**) additional filter of 0.3 mmCu.

The results of sensitivity as a function of effective energy are presented in Figure 6. The RPLDs had higher sensitivity than the Sn-RPLDs and OSLDs; however, the sensitivity was lower when the tin filters were applied to the RPLDs. The sensitivity of the OSLDs was similar to that of the Sn-RPLDs regardless of tube voltage or the thickness of the additional copper filter.

### 3.3. Directional Dependence

**Figure 7:**
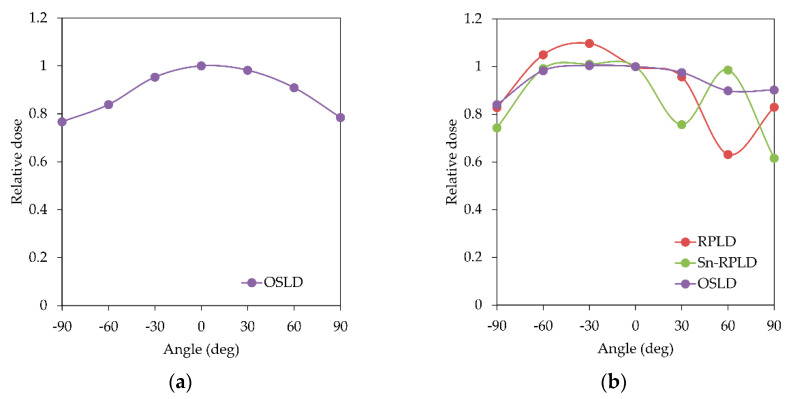
Relative dose values as a function of the dosimeter angle: (**a**) horizontal direction and (**b**) vertical direction.

The relative dose values as a function of the dosimeter angle are shown in Figure 7. The relative dose value of OSLD gradually decreased as the OSLD angle became greater or smaller than 0° in the horizontal direction. In the vertical direction, the relative dose values of RPLD, Sn-RPLD, and OSLD were in the range of 0.632–1.10, 0.616–1.01, and 0.840–1.00, respectively. The relative dose value of RPLD suddenly decreased at 60°, and Sn-RPLD suddenly decreased at −90°, 30°, and 90°.

### 3.4. Eye Lens Dose Measurement That Simulated an ERCP Physician

**Figure 8:**
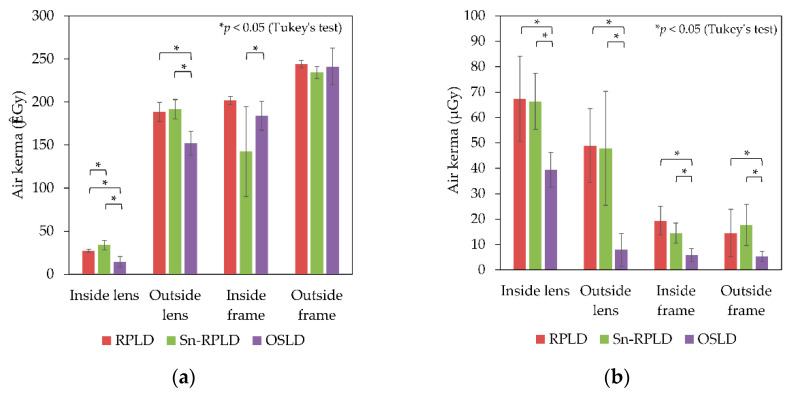
Air kerma obtained from small dosimeters pasted on the radioprotective glasses of the physician phantom: (**a**) left side and (**b**) right side.

The air kerma obtained from RPLDs, Sn-RPLDs, and OSLDs pasted on the radioprotective glasses of the physician phantom is shown in Figure 8.

Significant differences of air kerma were observed among the types of dosimeters on the inside of the glass, outside of the glass, and inside of the frame on the left side, which was nearer to the patient phantom (*p* < 0.05). The OSLDs showed significantly lower doses than the RPLDs and Sn-RPLDs on the right side, which were further from the patient phantom (*p* < 0.05).

In all types of dosimeters, the air kerma obtained from the left outside of the glass was higher than that obtained from the left inside of the glass. Similarly, air kerma obtained from the left outside of the frame was also higher than that obtained at the left inside of the frame. However, the air kerma obtained from the right outside of the glass was lower compared with that obtained from the right inside of the glass.

## 4. Discussion

The present study evaluated the property of small dosimeters used for measuring eye lens doses for medical staff during fluoroscopic examination. Dose linearity, energy dependence, and directional dependence on scattered X-rays as basic characteristics of small dosimeters were evaluated for RPLDs, Sn-RPLDs, and OSLDs by using overcouch radiographic and fluoroscopic X-ray equipment. In addition, air kerma obtained from these dosimeters, pasted on radioprotective glasses of the phantom simulating a physician who performed ERCP, were compared. The results of this study indicated that RPLDs, Sn-RPLDs, and OSLDs had different dosimeter properties, and affected the measured values of eye lens doses for the physician phantom during the fluoroscopic examination.

Previous studies showed that the response of RPLD, Sn-RPLD, and OSLD yielded a linear proportion to the radiation dose [12,17,18], and the current study indicated similar linear correlations between fluoroscopic time and air kerma accumulated from scattered X-rays in all types of dosimeters. A tendency was noted that the coefficients of variation for the accumulated air kerma measured by Sn-RPLDs were larger than those measured by RPLDs and OSLDs. Thus, the tin filters may increase the variation of accumulated air kerma because they generated scattered X-rays.

The present study showed that the energy dependence of Sn-RPLDs and OSLDs was smaller than that of RPLDs. The sensitivity of the RPLDs gradually changed (3.85–4.69) for 40.1–56.4 keV of effective energy in the present study, although the sensitivity curve of RPLDs had a peak at around 40 keV when irradiating monochromatic X-ray beams of 10–100 keV [19]. This difference may have been seen by the difference in the X-ray spectrum irradiated or scattered to the RPLDs. The scattered X-rays from the phantom were also polychromatic because polychromatic X-rays were irradiated to the acrylic block phantom in the present study. The sensitivity of the Sn-RPLDs was 0.872–1.30, which was similar to that of the OSLDs (0.963–1.33) regardless of the tube voltage and thickness of the additional copper filter. Therefore, tin filters are effective for compensating the energy dependence of RPLDs against not only primary X-rays but also scattered X-rays. However, sensitivity correction is crucial before use no matter which small dosimeters are chosen.

The difference in shapes between RPLD and OSLD elements was estimated to influence the results of directional dependence. The OSLD element formed into a disk shape, and the relative dose value of the OSLD was the highest when the direction of the scattered X-rays was perpendicular to the OSLD in both the horizontal and vertical directions and decreased gradually when the angle of the OSLD against the scattered X-rays was larger or lower than the right angle. Consequently, the angular dependence of the RPLD along the vertical direction only needs to be considered because the RPLD element formed into a cylindrical shape, and an understanding that the relative dose value of the RPLD suddenly decreased was needed when the RPLD was tilted to 60°, which was parallel to the direction of the scattered X-rays in the vertical direction. The tin filter was another factor for changing directional dependence, and the relative dose value of the Sn-RPLD suddenly dropped at three different angles (−90°, 30°, and 90°) in the vertical direction. Furthermore, Silva et al. [10] mentioned that attention must be given to the orientation of the Sn-RPLD because the scattered radiation coming from the patient can reach the eyes of the operator at high angles, underestimating or overestimating the dose received by the eye lens. Therefore, the tin filter was effective for compensating the energy dependence of RPLDs. However, it made the directional dependence more complicated when measuring scattered X-rays.

A previous study showed that the Dosiris eye lens dosimeter had good dose linearity, energy dependence, and angular dependence in the diagnostic X-ray energy domain [8]. Therefore, it could have better basic characteristics than RPLDs, Sn-RPLDs, and OSLDs for measuring occupational eye lens doses. It should also be considered that each type of small dosimeter has intrinsic advantages and disadvantages with respect to the system complexity, user-friendliness, and operator labor requirements [20].

In the dose measurement that simulated an ERCP physician, the air kerma obtained from OSLDs was significantly lower than those obtained from RPLDs and Sn-RPLDs except at the outside of the frame on the right side (*p* < 0.05, Tukey’s test). A possibility exists that the directional dependence of OSLDs along the horizontal axis may underestimate air kerma at those measurement points. However, further research is needed. The relative difference of air kerma between OSLDs and RPLDs (with and without tin filter) was especially larger on the right side, which was on the opposite side of the patient. Thus, the OSLDs placed on the right side against the scattered X-rays were estimated to be much larger or lower than the right angle compared with those on the left side. In addition, no significant difference was observed except on the left inside of the glass when air kerma was compared between RPLDs and Sn-RPLDs. Therefore, the influence of energy and directional dependence of RPLDs and Sn-RPLDs on the eye lens dose measurement that simulated an ERCP physician may small if the sensitivity is corrected beforehand to reduce the influence of energy dependence.

Previous studies evaluated scattered radiation dose in X-ray examination rooms using the “jungle gym” method, which uses OSLDs and a jungle gym apparatus consisting of paper pipes and plastic joints [21,22]. In the jungle gym method, the influence of directional dependence of OSLDs in the horizontal direction seems to be small because the OSLDs are pasted onto the plastic joints in the direction of the scattered X-rays. Although the influence of energy dependence and directional dependence in the vertical direction of the OSLDs is difficult to estimate from only our results, this study believes that the influence of directional dependence in the horizontal direction on the scattered radiation dose measured by the jungle gym method was smaller than that on the eye lens dose measured by the present study.

This study has several limitations. First, thermoluminescence dosimeters (TLDs), which have been used for a long time as passive radiation detection devices for dose monitoring or for measuring the dose affecting the patient, were not assessed in this study. Second, only one pair of radioprotective glasses, one location and direction of the physician phantom, and one set of irradiation parameters were used when simulating an ERCP physician to evaluate the property of small dosimeters used for measuring eye lens doses for the physician during ERCP. Finally, the influence of scattered X-ray energy spectra on the dosimetry accuracy according to dosimeter types was not evaluated. The scattered X-ray energy spectra changed according to the tube voltage, filter thickness, and scattering angle [10]. Although the energy dependence of RPLDs, Sn-RPLDs, and OSLDs was evaluated by changing the tube voltage and the thickness of an additional copper filter, further investigation is needed to clarify the influence of scattered X-ray energy spectra on the dosimetry accuracy of RPLDs, Sn-RPLDs, and OSLDs.

## 5. Conclusions

This study evaluated the property of RPLDs, Sn-RPLDs, and OSLDs used for measuring eye lens doses for medical staff during fluoroscopic examination. As basic characteristics of these small dosimeters, dose linearity, energy dependence, and directional dependence of scattered X-rays were evaluated using overcouch fluoroscopic X-ray equipment. In addition, air kerma obtained from these small dosimeters pasted on radioprotective glasses of the phantom simulating an ERCP physician were compared. The results showed that RPLDs, Sn-RPLDs, and OSLDs had different dosimeter properties, and they influenced measured values of eye lens doses for an ERCP physician, especially on the opposite side of the patient.

## Figures and Tables

**Table 1:** Effective energy at different tube voltages and added filtration settings.

Tube Voltage (kV)	Effective Energy (keV) ^1^	
	0.2 mmCu	0.3 mmCu
60	40.1	42.4
70	43.4	46.0
81	46.4	49.2
90	48.6	51.2
100	49.8	53.2
109	52.0	54.7
121	53.7	56.4

^1^ It was measured by using a RaySafe X2 R/F Sensor (Unfors RaySafe AB, Billdal, Sweden).

## Data Availability

Data sharing not applicable.
